# Utilization of Smartphone Depth Mapping Cameras for App-Based Grading of Facial Movement Disorders: Development and Feasibility Study

**DOI:** 10.2196/19346

**Published:** 2021-01-26

**Authors:** Johannes Taeger, Stefanie Bischoff, Rudolf Hagen, Kristen Rak

**Affiliations:** 1 Department of Otorhinolaryngology, Plastic, Aesthetic and Reconstructive Head and Neck Surgery University Hospital Würzburg Würzburg Germany

**Keywords:** facial nerve, facial palsy, app development, medical informatics, eHealth, mHealth, Stennert’s index, depth mapping camera, smartphone sensors

## Abstract

**Background:**

For the classification of facial paresis, various systems of description and evaluation in the form of clinician-graded or software-based scoring systems are available. They serve the purpose of scientific and clinical assessment of the spontaneous course of the disease or monitoring therapeutic interventions. Nevertheless, none have been able to achieve universal acceptance in everyday clinical practice. Hence, a quick and precise tool for assessing the functional status of the facial nerve would be desirable. In this context, the possibilities that the TrueDepth camera of recent iPhone models offer have sparked our interest.

**Objective:**

This paper describes the utilization of the iPhone’s TrueDepth camera via a specially developed app prototype for quick, objective, and reproducible quantification of facial asymmetries.

**Methods:**

After conceptual and user interface design, a native app prototype for iOS was programmed that accesses and processes the data of the TrueDepth camera. Using a special algorithm, a new index for the grading of unilateral facial paresis ranging from 0% to 100% was developed. The algorithm was adapted to the well-established Stennert index by weighting the individual facial regions based on functional and cosmetic aspects. Test measurements with healthy subjects using the app were performed in order to prove the reliability of the system.

**Results:**

After the development process, the app prototype had no runtime or buildtime errors and also worked under suboptimal conditions such as different measurement angles, so it met our criteria for a safe and reliable app. The newly defined index expresses the result of the measurements as a generally understandable percentage value for each half of the face. The measurements that correctly rated the facial expressions of healthy individuals as symmetrical in all cases were reproducible and showed no statistically significant intertest variability.

**Conclusions:**

Based on the experience with the app prototype assessing healthy subjects, the use of the TrueDepth camera should have considerable potential for app-based grading of facial movement disorders. The app and its algorithm, which is based on theoretical considerations, should be evaluated in a prospective clinical study and correlated with common facial scores.

## Introduction

Due to significant functional and cosmetic restrictions, facial palsy is a clinical symptom that is associated with a very high degree of psychological strain for those affected [1]. The incidence of peripheral facial palsy is 23-35 cases per 100,000 people; the incidence of the central form remains unknown. Both sexes are often affected equally, and those in the age groups of 30-50 years and 60-70 years are especially affected [2]. Affected patients can have severe functional problems ranging from asymmetry at rest, decreased facial movement, incomplete eye closure, problems eating and drinking, and a decreased sense of taste [3,4]. The fact that no underlying disease can be identified in about three-quarters of the cases (currently designated as idiopathic) also represents an unsatisfactory diagnostic situation [2]. There is still a great need for research in this area in order to ultimately be able to offer targeted therapy for these cases. Restoring facial function to the greatest possible extent can lead to improved self-confidence and a higher quality of life [5-7]. However, not only the diagnosis but also the quantification of facial palsy are still problematic.

### Current Methods for Quantifying Facial Palsy

Various systems have been established for the scientific and clinical classification of the degree of facial palsy, assessment of therapeutic interventions, and spontaneous disease course. These standardized systems facilitate effective communication with patients and professionals. The most widely established clinician-graded scoring systems are the Stennert index [8], House-Brackmann scale [9], and Sunnybrook Facial Grading System [10]. Numerous efforts to generally improve facial grading systems have been described in the literature. For example, an approach was published by Banks et al [11] with the electronic, clinician-graded facial function scale (eFACE), which was subsequently evaluated internationally [11-13]. Nevertheless, like other clinician-graded scoring systems, eFACE is also subject to bias and human error [14]. While a comparatively simple measuring method with a conventional hand ruler was propagated by Manktelow et al [15] in 2008, the immense technological progress of the past few years has led to software-based methods. Here, the measurement of facial expressions with Adobe Photoshop [16], for example, but also semiautomatic photographic assessment tools [17] were developed. Katsumi et al [18] described an advanced 3-dimensional (3D) facial motion measurement system, consisting of a color charge-coupled device camera and 2 laser scanners in combination with their proprietary software. In this experimental setting, the test subjects were asked to perform various facial movements. The authors describe that the measurement time was less than 5 minutes in each case. After the measurement, the data were transferred to a computer and then evaluated and compared with the clinical results from the Yanagihara and House-Brackmann grading scales, whereby a good correlation could be demonstrated [18]. Furthermore, several machine learning approaches [19-21] have been developed. Despite all efforts in the description and validation of various systems, none could achieve universal acceptance in everyday clinical practice. This is due to the fact that many systems are characterized as being very time-consuming or requiring expensive and complicated hardware, which is why some can hardly be used in routine practice [14].

Hence, it would be desirable to have a quick and precise tool for assessing the functional status of the facial nerve. This would improve and streamline the data situation for symptom severity in the case of facial palsy and the effectiveness of different treatment methods like the application of prednisolone or acyclovir [4]. In addition, it would enable patients without any required training to perform a reproducible measurement of the extent and course of their own disease, which might lead to an increase in motivation for performing therapeutic activities like facial exercises. For the latter, positive effects in the treatment of facial paralysis have been demonstrated [6,22-27].

### Smartphone Sensors as Sophisticated Medical Tools

Since the first smartphone was announced by Apple in 2007, the technical development of smartphones has progressed rapidly. In addition to an immense increase in computing power and storage options, the devices have been gradually equipped with high-precision sensors. For example, newer smartphone generations have an acceleration sensor, 3-axis gyroscope, barometer, proximity sensor, ambient light sensor, GPS, digital compass, microphone(s), and sophisticated camera system(s) [28]. In addition, high speed wireless communication platforms, such as long-term evolution, allow connection to health care providers at any time [29,30].

**Figure 1:**
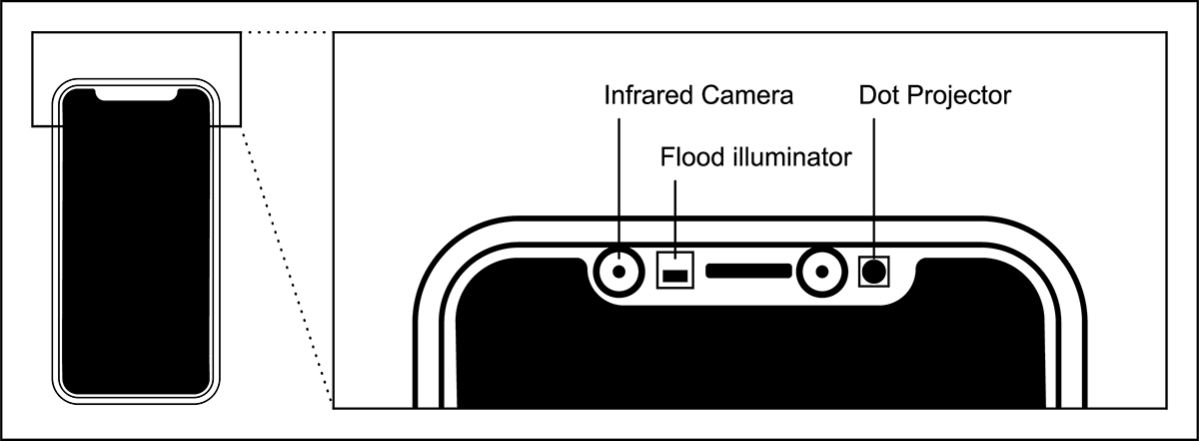
Hardware components of the TrueDepth camera system, which is integrated in the upper part of the smartphone. A Dot Projector throws over 30,000 infrared dots onto the face of the user, and the dots and an infrared image are captured via an infrared camera to create a depth map of the face (modified from [20]).

With regard to measurements of different facial muscles, one feature of recent smartphones is of high interest: the TrueDepth camera system. It enables user authentication via face recognition with the Face ID system and has been implemented since the iPhone generation X. The exact registration of facial data works via the projection of 30,000 infrared points, which are recorded at 60 times per second via an infrared camera and processed by a neural engine of the smartphone chip. An infrared flood illuminator adds infrared light to enable measurements also in the dark (Figure 1). An accurate 3D depth map of the face is calculated from the measurement data, which is converted together with the infrared image to a mathematical representation of the user’s facial features. According to the manufacturer, the technology works so precisely that the probability of false-positive detection of a random person is 1:1,000,000. In addition, the technology functions even with modifiers of the user’s appearance such as makeup or beard growth as well as hats, glasses, contact lenses, or scarves [31,32].

Apple’s ARKit framework enables developers to use face tracking with the TrueDepth camera for applications that go beyond Face ID. It is possible to access 52 predefined facial movement features, the extent of which is expressed in a numerical value ranging from 0.0 (neutral) to 1.0 (maximum movement) [33]. These features are also calculated 60 times per second, which results in a very high temporal resolution of the recorded facial movements.

The aim of the present study was to develop an app prototype that objectively and reproducibly quantifies facial asymmetries with the data from the iPhone’s TrueDepth camera system. With the developed app, it was possible to perform the measurement within a few seconds and without any additional external devices or the need for calibration. The extracted data were used to calculate a new index for the grading of unilateral facial palsy, called the Digital Facial Index (DFI), which is represented in a numerical value of 0%-100% for intuitive understanding.

### Conception of the App

During the content-related conception, the following features of the app were defined:

- Start screen with navigation elements and a setting option for the duration of the actual measurement to meet the needs of different users (for inexperienced users, for example, a time window for the measurement of 10 seconds might be too short, whereas for other users, 5 seconds is sufficient)
- Measuring mode with a simple instruction on 3 functionally relevant facial movements (“raise your eyebrows,” “close your eyes,” “pull up the corners of your mouth”) and a countdown timer over the previously set duration of the measurement
- Evaluation screen with an easily understandable representation of the individual measurement parameters and the calculated output of the newly developed index (DFI)
- Buttons to save the DFI measurement results within the app for simple statistics and repeating the measurement
- Raw data output for scientific evaluation

## Methods

### App Development

A native app architecture under iOS (Apple Inc, Cupertino, CA) was chosen to develop the app. One of the reasons why Apple devices and Apple’s ecosystem were chosen is that the Face ID technology, which is based on 3D data collection, is considered unrivalled compared to the 2-dimensional technology of its competitors [34]. In addition, iOS is primarily suitable for the development of medical apps due to its high security standards, compatibility, usability, and stability , even if it is not possible to prove these factors objectively in their full extent.

**Figure 2:**
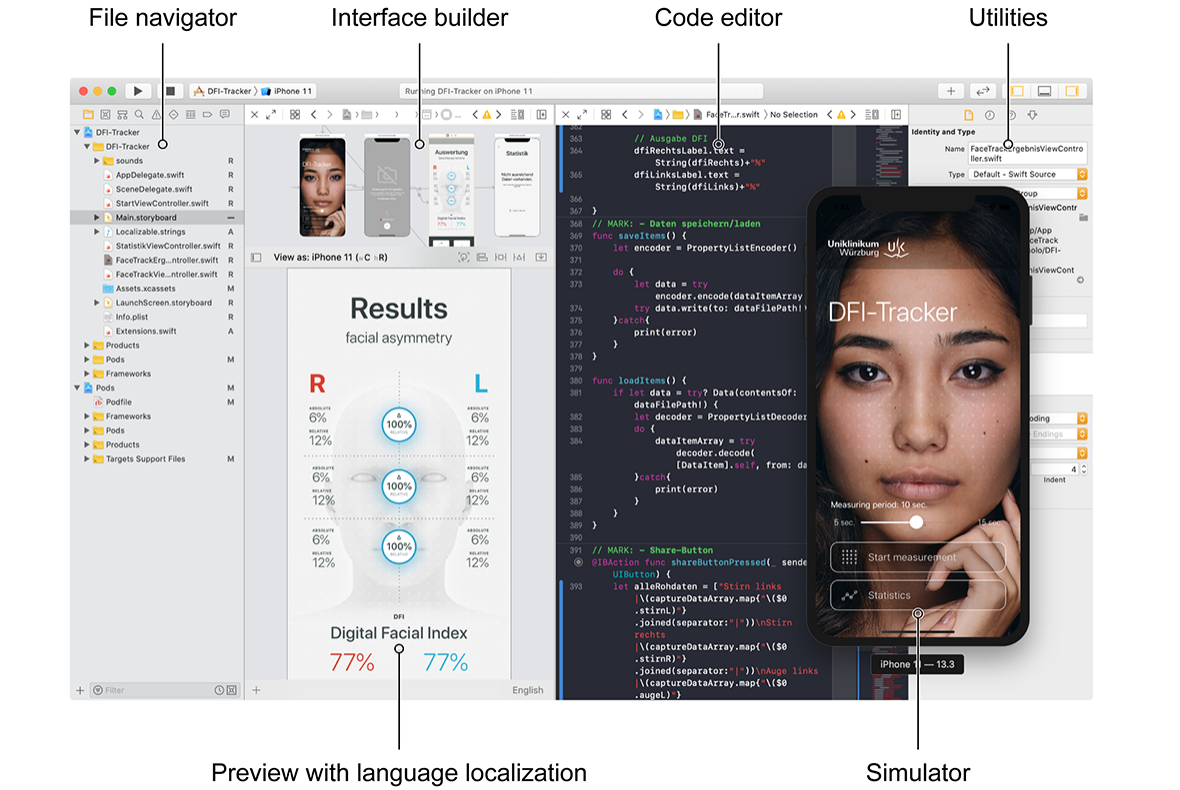
Screenshot of the app developed in the Apple Xcode Development environment. All associated project files are listed in the file navigator. The graphical user interface is designed with the interface builder and then linked to the code, which is written in the code editor. A utility area allows further settings. For the implementation of different regions, the user interface can be checked via the preview in the corresponding language. The simulator enables testing of the app on various virtual devices with the iOS operating system.

The user interface was designed with Adobe Creative Suite products (Photoshop 2020, Illustrator 2020; Adobe Inc, San José, CA) and the Sketch App (Version 61.2; Bohemian Coding, London, UK), which is specially optimized for the design of mobile apps. Then, the app was implemented using the Swift programming language in the latest version of the Xcode development environment (Apple Inc; Figure 2). An open-source library (ScrollableGraphView [35]) was integrated with the dependency manager CocoaPods [36] to graphically display the stored data in the form of a graph. As far as possible with a prototype, the Mobile App Rating Scale was used as a template for compliance with established quality criteria [37].

### Calculation of the Digital Facial Index

**Figure 3:**
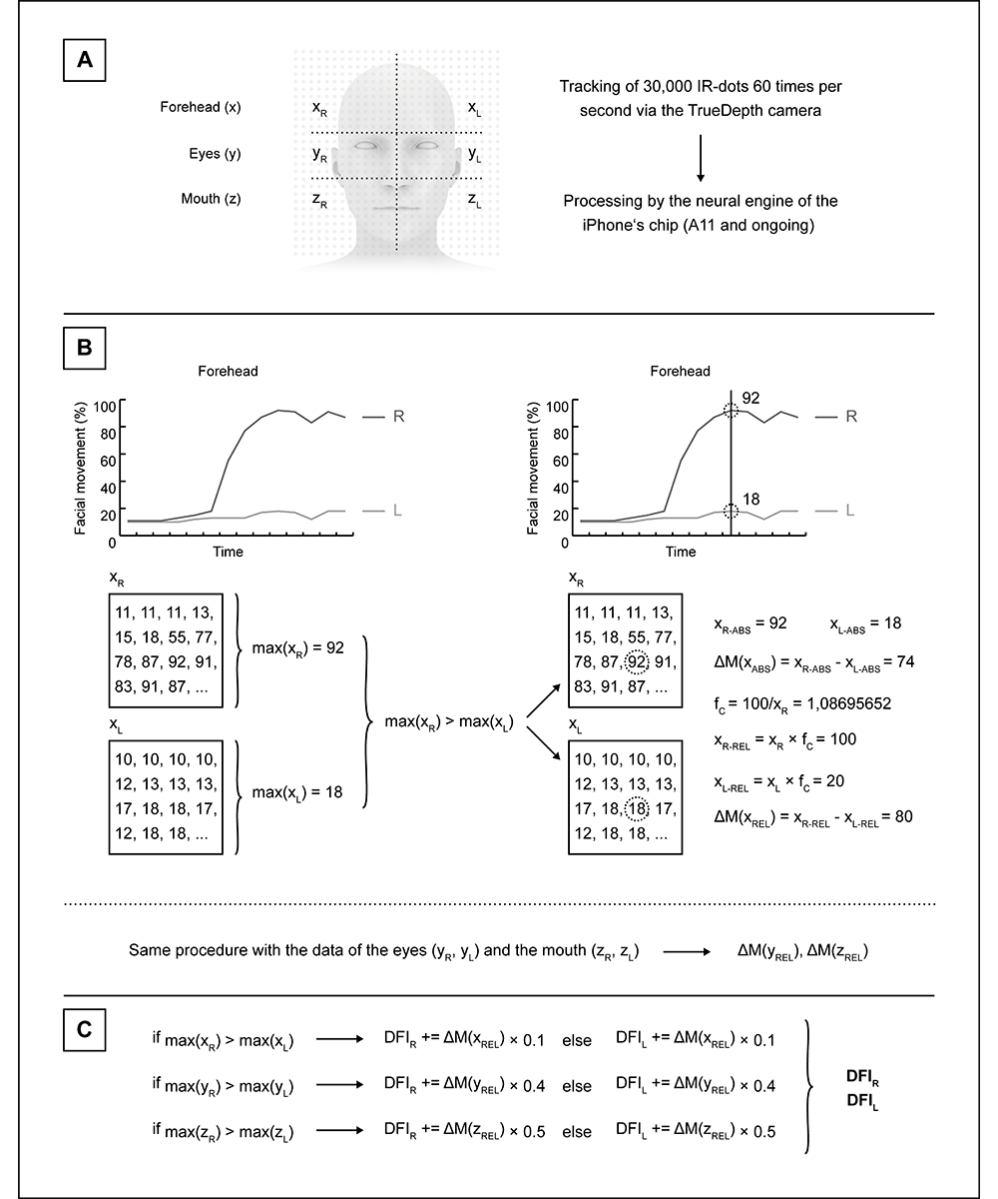
Algorithm for the calculation of the Digital Facial Index (DFI): (A) The TrueDepth camera tracks 30,000 infrared dots projected by the iPhone’s dot projector, and the data are rendered as so-called blend shape coefficients for the (B) measurement time window for the forehead, eyes, and mouth (exemplary data shown), and the maxima of both sides are compared for each facial region; the half of the face with the larger maximum corresponds to 100% (relative value). (C) The DFI is calculated by adding the relative differences (∆M based on absolute and relative values) of the 3 facial regions, with a weighting, similar to Stennert´s index.

Since the data output for the specific facial features via the ARKit framework has a value ranging from 0.0 to 1.0, the expression of the movement extent in the form of an easily understandable percentage value seemed rational. Similar to the description by Stennert et al [8], individual facial regions should be matched with a defined weighting based on functional and cosmetic aspects, namely with the proportions of 10% for the forehead, 40% for the eye area, and 50% for the mouth region. The newly developed DFI should adhere exactly to this weighting for optimal follow-up correlation using the Stennert index that applies an easily comparable scaling (0-10). The algorithm for calculating the DFI is shown in Figure 3: The so-called blend shape coefficients, which describe the movement of the eyebrows, closure of the eyelids, and upward movement of the corners of the mouth (in accordance with the Apple Developer Documentation) are recorded at 60 times per second and saved as an array. From this data record, the respective maximum for the individual feature is determined. For each facial region (forehead: x; eyes: y; mouth: z), it is assumed that in a patient suffering from unilateral facial palsy, the side with the greater maximum corresponds to the healthy side. The numerical value of the facial movement of the opposite side in each specific region is used to compare with the maximum of the stronger (healthy) side. The difference in facial motility ΔM(x_ABS_, y_ABS_, z_ABS_) for the 3 regions is calculated by subtracting these 2 numerical values. As it was observed in test runs with the prototype in healthy subjects, the respective absolute values ​​often do not total 100% despite a complete raising of the eyebrows, a full closure of the eyelids, and a maximal lifting of the corners of the mouth; therefore, a correction factor f_C_ was introduced. This scales the value of the half of the face with higher values in 1 of the 3 areas to 100% and the half of the face with the lower values proportionally. This aims to obtain a realistic evaluation of facial symmetry, derived from theoretical considerations. Thus, relative values ​​and the corresponding relative differences ΔM(x_REL_, y_REL_, z_REL_) are calculated, which are ultimately used in the calculation of the DFI. For the 3 regions, the relative differences are summed up for the left and the right depending on the identified side of facial palsy by comparison of the maximum values for each region. A patient affected by facial palsy will initially have higher differences calculated in this way in terms of facial asymmetry. In most patients, these differences become smaller [38]. In order to represent the course of the disease in a positive trajectory, the maximum extent of the facial palsy was set to 0% and a perfectly symmetrical face to 100%. Graphically represented, an increasing rather than a decreasing curve should result in most cases. Thus, this procedure is reciprocal to Stennert´s index, in which the highest value (10) corresponds to complete facial palsy.

### Data Display and Sharing Options

Following a successful measurement, a screen opens automatically displaying the analyzed data. It shows all calculated values of the 3 facial regions including the resulting DFI values. There are also buttons for repeating the measurement and storing the current data, as well as a button that enables raw data output for scientific purposes. This was implemented using the *UIActivityViewController* class from Apple's UIKit, which allows the sharing of data (eg, via AirDrop, email, or text message).

### Test Measurements

Applying the prototype developed as described in the previous section, all 4 authors of this publication carried out 10 consecutive self-measurements using an iPhone X_R_ to evaluate the intertest variability. In this setting, all test subjects looked straight ahead into the smartphone's front camera or display while holding the device in their hands.

To check the robustness under unfavorable conditions, the results were compared with those obtained from one individual to determine whether different angles of the smartphone front camera relative to the face (reference point: median of the head at eye level; distance: 37.5 cm, which is within the distance of 25-50 cm recommended by the manufacturer and roughly corresponds to a natural holding position) leads to different results. During this measurement, the positions of both the smartphone and the subject's face were fixed with a tripod. In addition, we checked whether incorrect measurements occur if the smartphone is held deliberately and intermittently leaves the camera's field of view.

No ethic committee approval at a named institution was needed and applied for, since only data from the authors were used. All authors provided written informed consent for their data and pictures used in the study.

Statistical analysis was performed using one-way analysis of variance in GraphPad Prism (Version 8.2.1; GraphPad Software Inc, San Diego, CA). Differences were considered significant when *P*≤.05.

The raw data of the measurements were imported as a text file in Microsoft Excel (Version 16.34; Microsoft Corporation, Redmond, WA) for rendering as a graph and to double check that the calculations within the app were correct. The measurements were also partially documented in the form of a screen recording with QuickTime Player (Version 10.5; Apple Inc) and the iPhone connected to a Mac computer (Apple Inc) using a lightning cable.

**Figure 4:**
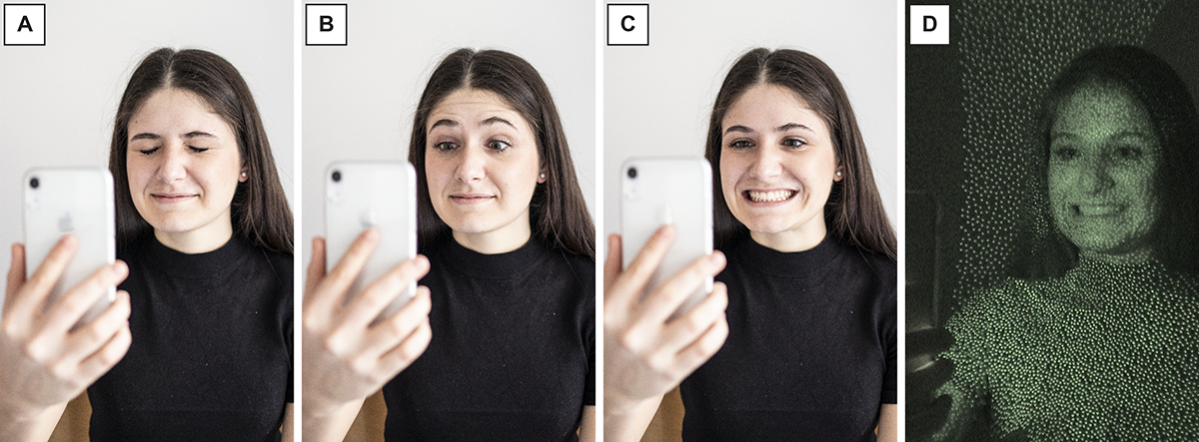
Test sequence using the Digital Facial Index (DFI) tracker app, with conventional photos during which the user performed 3 facial movements during a defined measurement time window, including (A) “close your eyes,” (B) “raise your eyebrows,” and (C) “pull up the corners of your mouth,” and (D) an infrared photograph that shows part of the 30,000 points thrown at the user by the smartphone’s dot projector to capture depth data of the face. The depicted author provided written informed consent for her photos to be used for this paper.

For demonstration purposes, conventional photographs of test sequences were taken with a Canon 80 D digital single-lens reflex camera (Canon Inc, Tokyo, Japan), with a Canon 35 mm f2.0 lens attached (Figures 4A-4C). In addition, an infrared image was recorded with a Sony DCR-TRV25E Camcorder (Sony Corporation, Tokyo, Japan) in “NightShot” mode during the measurement (Figure 4D).

## Results

On the basis of the described theoretical considerations, a functioning, stable app prototype could be developed that utilizes the TrueDepth camera system and the ARKit framework to measure the extent of facial motions fast and objectively. In the latest version of the prototype, no runtime nor buildtime errors occurred. The requirements for the app that were initially defined could be fully met.

**Figure 5:**
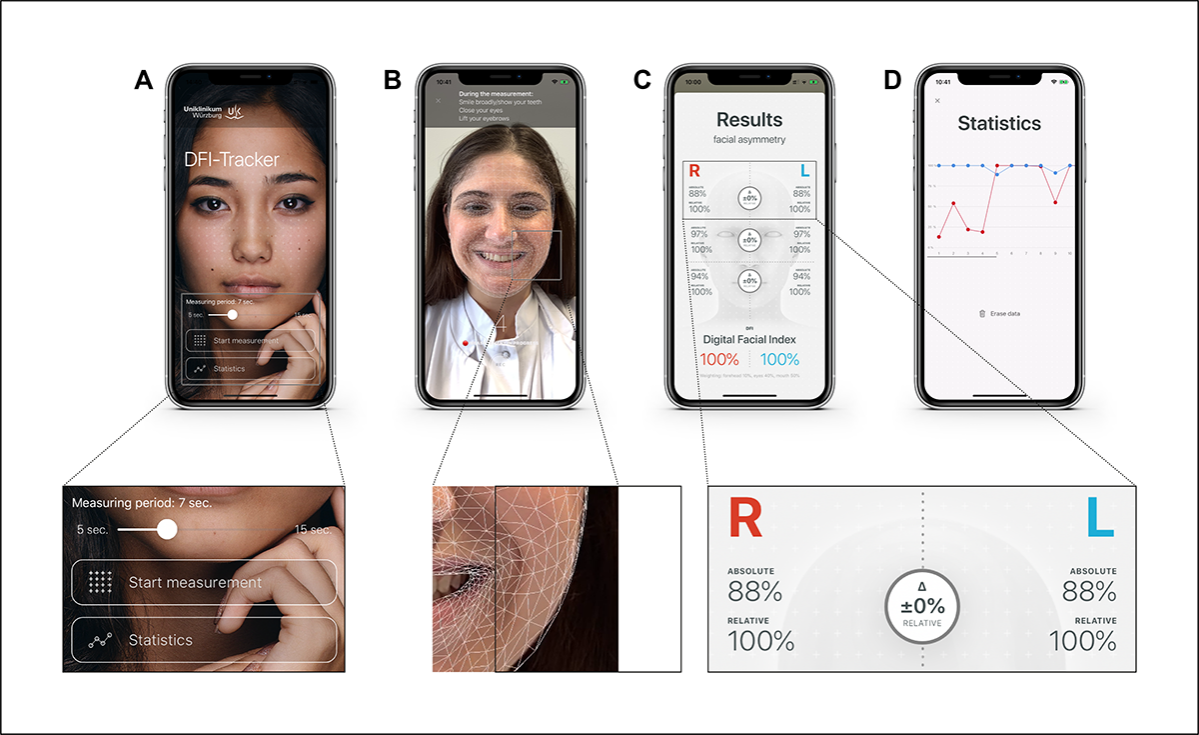
User interface of the Digital Facial Index (DFI) Tracker App, including the (A) start screen with a setting for the measurement duration (5 to 15 seconds) and buttons to get to the measurement mode or statistics; (B) measurement mode with a real-time polygon grid overlay for immediate feedback on the precise registration of the user’s face; (C) results showing absolute and relative values, their difference, and calculated DFI values; and (D) statistics with a simple graphical representation of the last measured values. The depicted author provided written informed consent for her photos to be used for this paper.

The complex underlying technology was successfully integrated into a user-friendly interface (Figure 5; Multimedia Appendix 1). When using the measurement mode, a real-time overlay of a polygon grid over the user’s face provides immediate feedback on the precise registration of the face.

With the newly defined DFI, the measurement result was an easily understandable numerical value expressed as a percentage for each half of the face. After a brief explanation (<1 minute; the authors were initially not exactly familiar with the measuring principle of the app prototype), measurement with the app prototype was easily and independently carried out by the respective test person within a time window of less than 10 seconds each (Multimedia Appendix 1). For scientific analysis, the raw data can be easily transferred to a computer via the “share raw data” button as a text file. The import into Microsoft Excel, for example, for statistical analysis was also an easy and efficient process due to the text file’s raw data structure, which was optimized for this purpose.

**Figure 6:**
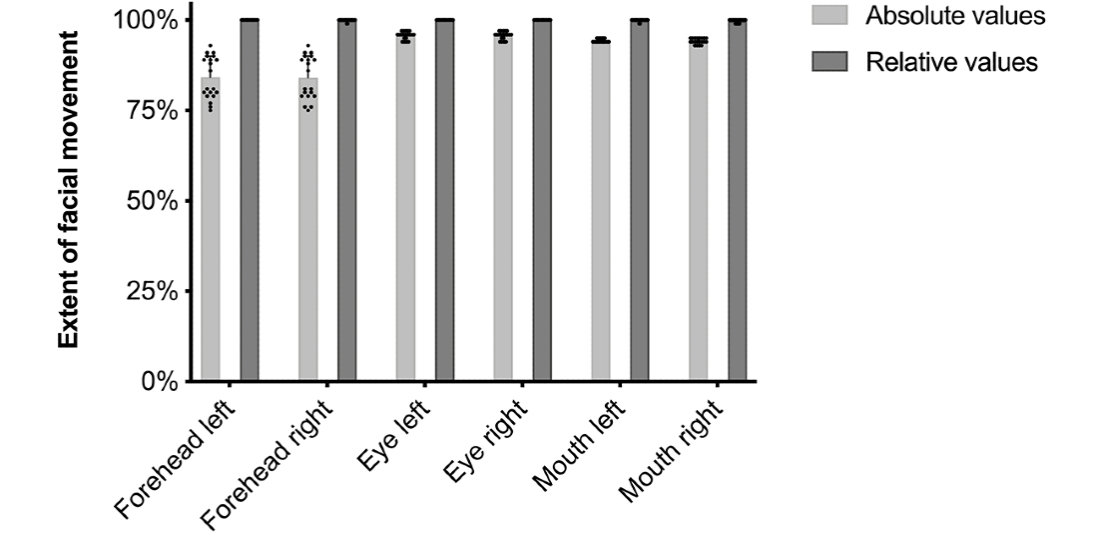
Intertest variability and SD (bars) of measurements with the app prototype and 4 healthy subjects (10 measurements each); no asymmetry of facial motor skills was found.

The measurements were reproducible and showed no statistically significant intertest variability (Figure 6; Multimedia Appendix 2). The DFI was ≥99% for both sides in all measurements and in all subjects with just one exception in a single measurement (97% on one side). Hence, all healthy test persons were identified correctly as having no facial movement disorder.

**Figure 7:**
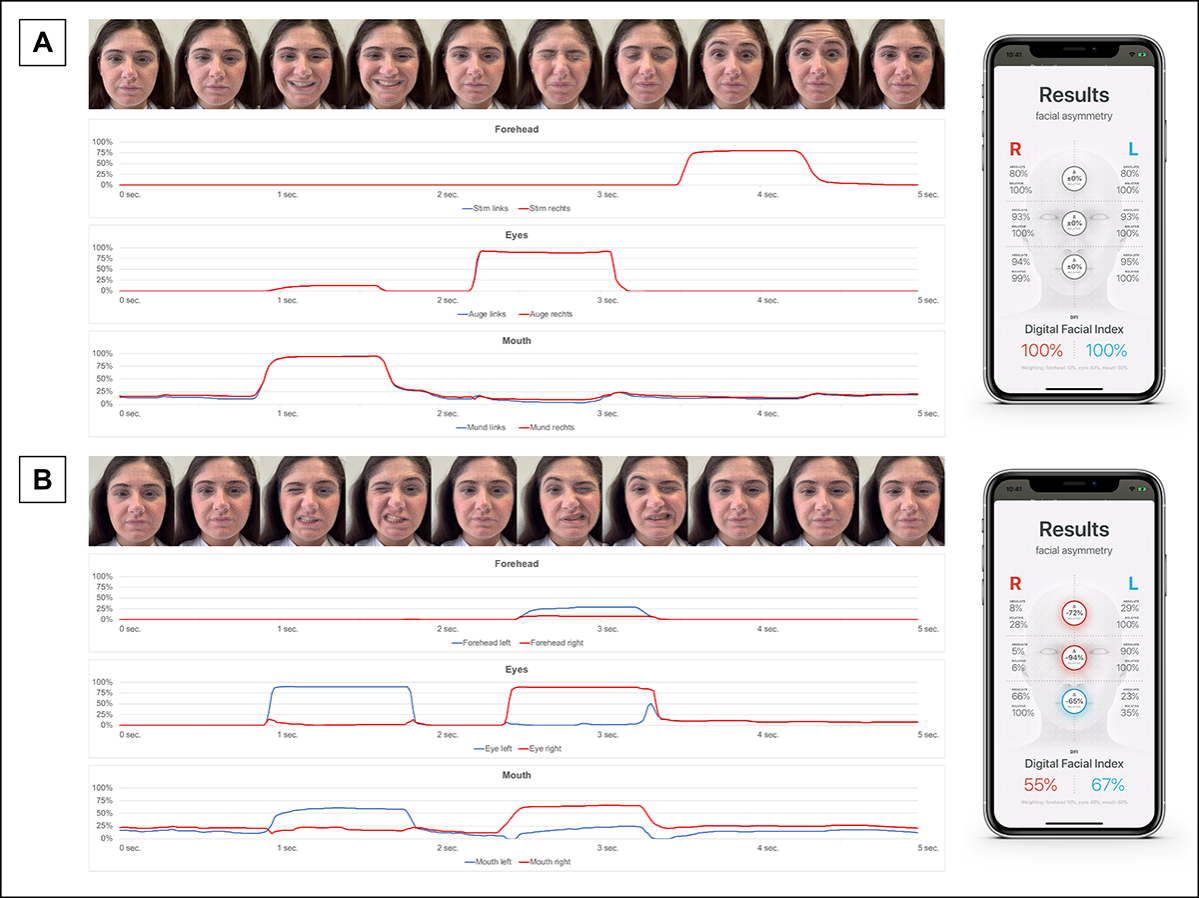
Test measurements with one of the (healthy) authors, showing the raw data (absolute values) over time, which are recorded 60 times per second, for a (A) normal measurement sequence, in which the test subject makes 3 grimaces that result in symmetrical facial motor skills as Digital Facial Index (DFI) values of 100% for both sides, and (B) simulation of asymmetrical facial features alternately for each side. The video stills shown here are mirrored horizontally, as is usual when shooting with the front camera of smartphones. The depicted author provided written informed consent for her photos to be used for this paper.

When comparing the calculated absolute and relative values and the resulting DFI values, there were no discrepancies in the visual comparison with the video recordings and the curves derived from the raw data (Figure 7A). Simulating facial asymmetries by grimacing also gave consistent results (Figure 7B). It should be noted here that a healthy test person is not able to correctly simulate all aspects of facial palsy. With maximum voluntary movement of the facial muscles on one side, the opposite side also moves to a small extent. In the example shown, our subject also found it more difficult to raise the right eyebrow as high as the left as part of the movements combined here. This results in a barely noticeable deflection of the absolute measured values for the right eyebrow.

Nevertheless, there is a symmetrical lifting of the eyebrows with higher measured values during the regular measurement process (Figure 7A). Manually checking the app's internal calculations with the raw data in Microsoft Excel did not identify any detectable errors.

**Figure 8:**
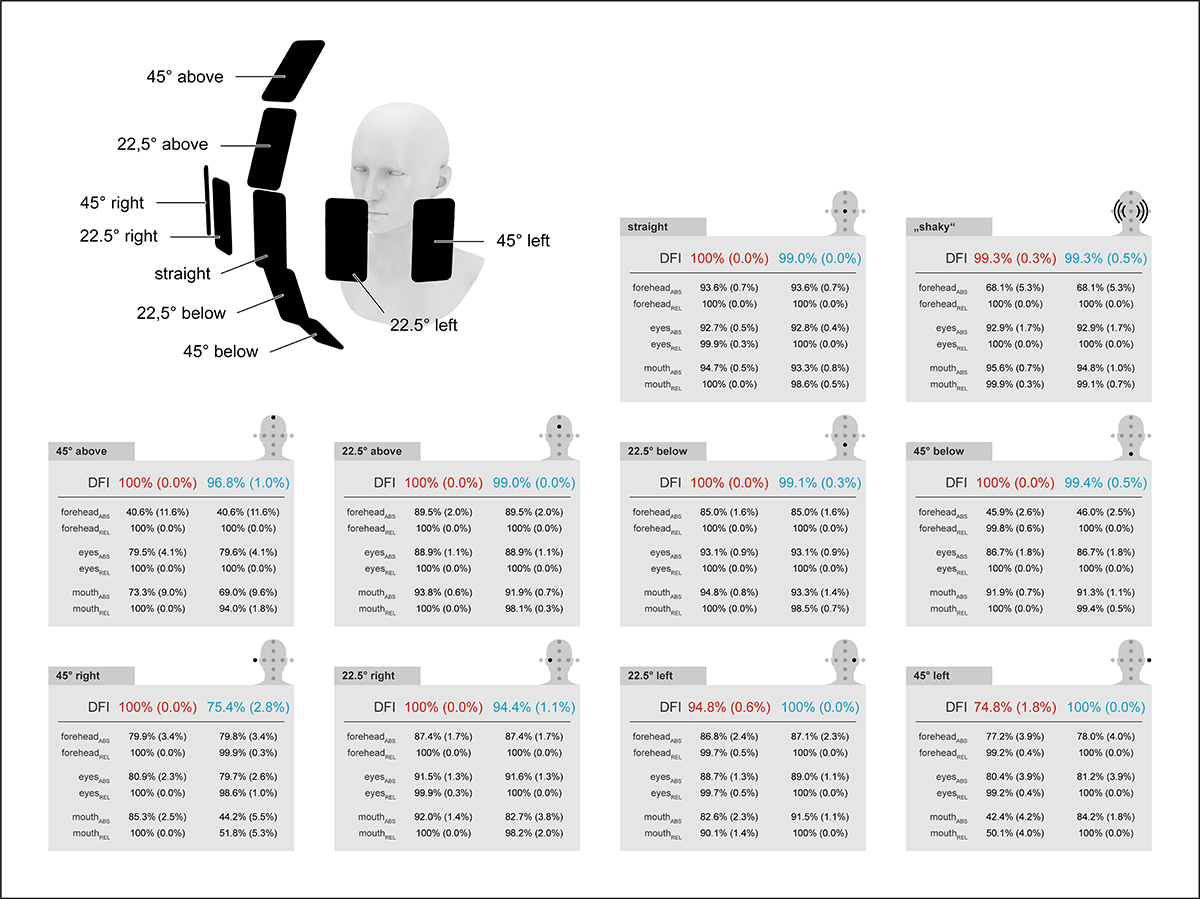
To simulate unfavorable measurement conditions, 10 measurements (mean [SD] shown) were carried out on a (healthy) test person at different angles relative to the median of the head at eye level and at 37.5 cm, with tripods to fix the position of the smartphone and the head of the test person; in addition, a measurement was made with deliberately shaky hand movements and occasionally leaving the face out of the camera's field of view.

The measurement from different angles (Figure 8) relative to the face gave consistent results when looking straight into the camera (DFI_R_ 100% [SD 0.0%], DFI_L_ 99.0% [SD 0.0%]), 22.5 ° from above (DFI_R_ 100% [SD 0.0%], DFI_L_ 99.0% [SD 0.0%]), 22.5 ° from below (DFI_R_ 100% [SD 0.0%], DFI_L_ 99.1% [SD 0.3%]), and 45 ° from below (DFI_R_ 100% [SD 0.0%], DFI_L_ 99.4% [SD 0.5%]). The measurements with a lateral deviation of 22.5 ° resulted in side differences for the opposite half of the face: The measurements 22.5° from the right resulted in DFI_R_ of 100% (SD 0.0%) and DFI_L_ of 94.4% (SD 1.1%) and 22.5° from the left resulted in DFI_R_ of 94.8% (SD 0.6%) and DFI_L_ of 100.0% (SD 0.0%). There were significant deviations at angles of 45 ° from above (DFI_R_ 100% [SD 0.0%], DFI_L_ 96.8% [SD 1.0%]) and 45 ° from the right (DFI_R_ 100% [SD 0.0%], DFI_L_ 75.4% [SD 2.8%]) and the left (DFI_R_ 74.8% [SD 1.8%], DFI_L_ 100% [SD 0.0%]). Interestingly, with these large angles, the mouth component is decisive for the measurement error, whereas no relevant errors occur for the forehead and eye regions. If measurements were made with deliberately shaky hand movements and occasionally leaving the face out of the camera's field of view, there were no relevant measurement errors (DFI_R_ 99.3% [SD 0.3%], DFI_L_ 99.3% [SD 0.5%]).

## Discussion

Smartphones, tablets, and related widely used devices such as smartwatches offer immense potential for the development of medical applications thanks to highly advanced sensors and computing power, as well as high storage capacities. For health monitoring and diagnosis, smartphone sensors have already proven to be highly potent tools in many medical specialties. To name just a few examples, smartphones are suitable for measuring heart rate and its variability [39-41]. Even the accurate automated detection of atrial fibrillation is possible [42-44]. For pulmonary medicine, low-cost spirometers were developed using the microphones of smartphones [45,46]. Smartphone cameras are suitable for retinal fundus imaging for assessing ophthalmic diseases [47-49], as well as for laboratory applications, like the detection of specific DNA sequences in combination with specific assays [50,51]. Other applications are for skin health monitoring [52-55], detection of anemia [56], sleep monitoring [57-59], and mental health assessment [60-66].

In addition, smartphones are highly available worldwide and are an integral part of everyday life. In September 2019, the number of active smartphones reached 3.6 billion. Although this work only describes an implementation for iPhones, which limits the potential target group to Apple's smartphone market share of 22.3% [67], a comparable solution for iPads and Android devices would be technically easy to implement. It is currently assumed that other manufacturers such as Samsung will also integrate a similar depth mapping camera system in future smartphones [68].

Regarding possible security concerns with respect to the TrueDepth data, Apple states that the data are protected by the so-called Secure Enclave (a coprocessor that provides cryptographic operations for data security) and do not leave the device or are backed up in an online storage medium without user permission [31,69]. The measurement results collected in the app prototype described are also not assigned to any user data nor are saved in a cloud without any active user action (eg, via the button for raw data export that is currently intended for further scientific analysis). In a future user version, features such as integration into electronic patient records would be desirable while maintaining correspondingly high security standards.

One limitation of this study is the fact that the newly developed app is only aimed for patients with unilateral facial palsy. However, bilateral facial palsy is very rare, with an annual incidence rate of 1:5,000,000 and only accounts for 0.3%-3.0% of all cases with facial palsy [70-72].

Selecting 3 of 52 possible facial features that can be extracted simultaneously using ARKit was intended to test movements that are also carried out when the motility criteria of Stennert’s index are assessed. The presence of facial synkinesis as well as the resting tone are not determined using the method described. However, as Stennert et al [8] stated in their description of the Stennert index in 1977, motility reveals more about the degree of residual innervation or reinnervation of the facial nerve than resting tone.

The formulas described here for interpreting the TrueDepth data and for calculating the DFI were created on the basis of theoretical considerations and aim for a symmetry of the facial motor skills by matching the face in 3 areas. Since the range of motion of the healthy half of the face—with the idea of symmetry as the ideal state—represents a reasonable measure for the sick side, the absolute values of the healthy side are scaled to 100% and compared with the proportionally scaled opposite side. However, it has to be stated that this approach was not chosen to achieve ultimate scientific accuracy, but to allow for a simple and realistic evaluation of facial symmetry. In combination with the existing functionality of the TrueDepth camera, there is no need for calibration. At present, it is not possible to estimate to what extent the newly designed app reflects the “reality” in patients with facial palsy and the course of their disease, which is currently expressed by established clinician-graded scoring systems. Nevertheless, based on the first experiences with the app prototype and with no statistically significant intertest variability in healthy subjects, it seems possible to advance a precise classification of the severity of facial palsy by the use of this app.

If you compare our approach with clinical scoring systems such as the Stennert Index, House-Brackmann scale, Sunnybrook Facial Grading System, or eFACE, we see our method clearly having an advantage in terms of intraobserver and interobserver variability and the duration of the measurement (a few seconds for the DFI measurement compared to a few minutes such as when collecting the 19 eFACE parameters). When compared with technical solutions, the solution described by Katsumi et al [73] has broad similarities in terms of the functional principle. However, this requires a relatively complex hardware setup. Since smartphones are ubiquitous and global and area-wide distribution of software via app stores is possible, we consider app-based solutions as described here to be much more practical and cheaper. The, from our experience, simple, fast, and practically self-explanatory measuring process should also make it possible for laypeople to measure themselves for self-assessment.

The DFI measurements from different angles and with deliberately shaky hands show that the measurement method is sufficiently robust in realistic scenarios. Since the user, from the experience of our test runs, intuitively looks into the camera during the measurement so that his face is completely shown in the display, relevant lateral axis deviations and the associated measurement errors are unlikely. Nonetheless, in a future end-user solution, it could make sense to provide the user with appropriate information about the correct measurement process.

Another positive aspect of the measurement method is that changes in external appearance, such as facial hair or glasses, should not have any effect on the results [32,33]. Weight increases or decreases in the time interval in which measurements of facial symmetry are made (eg, 6 months) should not have any relevant effects, except in extreme cases, due to the pure assessment of the maximum range of facial movements.

On the other hand, acquired or congenital asymmetries such as scars, deformities, or grafts on the face could of course contribute to a relevant falsification of the measurable range of motion. However, these can also influence established rating systems such as the Stennert’s index, based on our everyday clinical experience.

The decision to configure the DFI reciprocal to Stennert´s index was based on clinical considerations. Following a regular course of healing, an increasing numerical value is graphically represented as an increasing curve. Although this is only a small detail, maybe it can contribute to a positive psychological effect for those affected.

The possibilities of the TrueDepth camera in everyday life, such as authorization of payments or unlocking of the mobile phone, are very popular among users. However, it is currently not possible to anticipate whether mobile phone manufacturers might cease the integration of depth mapping cameras into its products or replace it by another technology. Nevertheless, the fact that Apple is currently considering integrating TrueDepth technology into notebooks [74] makes it likely that TrueDepth cameras will also be installed in future device generations. In general, the focus in Apple's marketing concept has recently been placed intensely on privacy. FaceID, which is based on TrueDepth camera technology, is an integral part of this concept [75]. In addition, even rear-facing depth mapping cameras may be added to enable improved augmented reality applications, for example. To what extent such a camera would then also be suitable for facial measurements (eg, by the physician at the patient’s bedside for better documentation of the facial nerve status) is currently unclear. In summary, the medical application described here should therefore also be feasible in the future.

With the app prototype described in this work, a highly precise, easy-to-use solution for the grading of facial movement disorders via the 3D-based measurements of the TrueDepth camera system was successfully designed. It does not require any expert knowledge to operate and delivers reproducible, easy-to-understand results within a few seconds. This empowers patients suffering from facial palsy to independently determine the progress of their therapy for this functionally and cosmetically severely impairing disease. The necessary hardware is widely available, and no external equipment is needed. Nevertheless, to our knowledge, this is the first time that the TrueDepth camera system has been used for this type of medical usage.

A prospective clinical study is necessary to check the validity by correlation with established clinician-graded scoring systems and to objectively assess the usability of the app. Depending on the result of such a study, the underlying algorithm might have to be adjusted to achieve an optimal fit. The app-based design enables large-scale, decentralized data collection for multicenter clinical studies, which can advance different fields in medical science.

In addition, the export of raw data with a temporal resolution of 60 times per second opens the interesting possibility of further scientific evaluation and development of other medical fields of application. It is conceivable that the evaluation of TrueDepth data could be used for purposes such as facial training methods with real-time biofeedback or the surveillance of dysfunctions of facial movement associated with conditions such as Parkinson’s disease, where spontaneous facial expressions appear to be selectively affected [76], or motor tic disorders with facial affection [77]. Automated, fast, objective tracking of facial motor skills could generate valuable data on the individual course of these diseases and on the response to different treatments.

Another possible application area would be in studies of affective psychology. For example, depressed individuals show fewer Duchenne smiles and less facial animation. This implies that automated facial analysis may prove to be useful in mental health screening too [78]. In a similar context, a smartphone-based solution has already been demonstrated with the Loki app as a proof-of-concept, which was developed in a Canadian hackathon project. By training a simple neural network with two hidden layers, it was able to map TrueDepth facial data to 4 emotions in real-time (happy, sad, angry, surprised) [79].

Furthermore, a comparable integration of machine learning into the current algorithm might even enable automated stroke screening using a smartphone in the future, as facial palsy is a very common consequence of strokes [80].

## Appendix

Multimedia Appendix 1 Exemplary measurement sequence with the app prototype. After the measurement, the absolute and relative values for the different facial regions as well as the calculated DFI score are displayed. The time course of the DFI scores can be shown in a diagram. The depicted author provided written informed consent for the video to be used for this paper.

Multimedia Appendix 2 Absolute and relative values measured and calculated by the app prototype and four healthy subjects.

## Abbreviations

3D: 3-dimensional
DFI: Digital Facial Index
eFACE: electronic, clinician-graded facial function scale
