# Supplementary material for: Utilization of Smartphone Depth Mapping Cameras for App-Based Grading of Facial Movement Disorders: Development and Feasibility Study
Source: JMIR Mhealth Uhealth. 2021 Jan 26;9(1):e19346. doi: 10.2196/19346 (PMC7872839; doi:10.2196/19346)
Supplement: Multimedia Appendix 2 [file mhealth_v9i1e19346_app2.docx]

**Supplementary Table 1:** Absolute and relative values measured and calculated by the app prototype and four healthy subjects.

|  | absolute values | | | | | | relative values | | | | | | DFI | |
| --- | --- | --- | --- | --- | --- | --- | --- | --- | --- | --- | --- | --- | --- | --- |
| subject | **FH_L_** | **FH_R_** | **E_L_** | **E_R_** | **M_L_** | **M_R_** | **FH_L_** | **FH_R_** | **E_L_** | **E_R_** | **M_L_** | **M_R_** | **DFI_L_** | **DFI_R_** |
| JT | 89 | 89 | 96 | 96 | 94 | 94 | 100 | 100 | 100 | 100 | 100 | 100 | 100 | 100 |
|  | 86 | 86 | 96 | 96 | 94 | 93 | 100 | 100 | 100 | 100 | 100 | 99 | 100 | 99 |
|  | 88 | 88 | 97 | 97 | 94 | 94 | 100 | 100 | 100 | 100 | 100 | 100 | 100 | 100 |
|  | 89 | 89 | 94 | 94 | 94 | 95 | 100 | 100 | 100 | 100 | 99 | 100 | 99 | 100 |
|  | 91 | 91 | 97 | 97 | 94 | 93 | 100 | 100 | 100 | 100 | 100 | 99 | 100 | 99 |
|  | 90 | 90 | 96 | 96 | 94 | 94 | 100 | 100 | 100 | 100 | 100 | 100 | 100 | 100 |
|  | 93 | 93 | 95 | 95 | 95 | 95 | 100 | 100 | 100 | 100 | 100 | 100 | 100 | 100 |
|  | 91 | 91 | 96 | 96 | 95 | 95 | 100 | 100 | 100 | 100 | 100 | 100 | 100 | 100 |
|  | 90 | 90 | 96 | 96 | 95 | 95 | 100 | 100 | 100 | 100 | 100 | 100 | 100 | 100 |
|  | 89 | 89 | 95 | 95 | 94 | 94 | 100 | 100 | 100 | 100 | 100 | 100 | 100 | 100 |
| SB | 75 | 75 | 96 | 96 | 95 | 95 | 100 | 100 | 100 | 100 | 100 | 100 | 100 | 100 |
|  | 77 | 76 | 94 | 94 | 94 | 95 | 100 | 99 | 100 | 100 | 99 | 100 | 99 | 100 |
|  | 80 | 80 | 94 | 94 | 95 | 95 | 100 | 100 | 100 | 100 | 100 | 100 | 100 | 100 |
|  | 81 | 81 | 96 | 96 | 95 | 95 | 100 | 100 | 100 | 100 | 100 | 100 | 100 | 100 |
|  | 79 | 79 | 96 | 96 | 94 | 94 | 100 | 100 | 100 | 100 | 100 | 100 | 100 | 100 |
|  | 79 | 79 | 97 | 97 | 94 | 94 | 100 | 100 | 100 | 100 | 100 | 100 | 100 | 100 |
|  | 81 | 81 | 94 | 94 | 94 | 93 | 100 | 100 | 100 | 100 | 100 | 99 | 100 | 99 |
|  | 76 | 76 | 96 | 96 | 95 | 94 | 100 | 100 | 100 | 100 | 100 | 99 | 100 | 99 |
|  | 80 | 80 | 97 | 97 | 94 | 94 | 100 | 100 | 100 | 100 | 100 | 100 | 100 | 100 |
|  | 80 | 79 | 97 | 97 | 94 | 93 | 100 | 99 | 100 | 100 | 100 | 99 | 100 | 99 |
| KR | 92 | 92 | 91 | 91 | 87 | 88 | 100 | 100 | 100 | 100 | 99 | 100 | 99 | 100 |
|  | 92 | 92 | 94 | 94 | 89 | 90 | 100 | 100 | 100 | 100 | 99 | 100 | 99 | 100 |
|  | 92 | 92 | 95 | 95 | 86 | 87 | 100 | 100 | 100 | 100 | 99 | 100 | 99 | 100 |
|  | 91 | 91 | 93 | 93 | 87 | 88 | 100 | 100 | 100 | 100 | 99 | 100 | 99 | 100 |
|  | 92 | 92 | 93 | 93 | 87 | 88 | 100 | 100 | 100 | 100 | 99 | 100 | 99 | 100 |
|  | 92 | 92 | 94 | 94 | 85 | 86 | 100 | 100 | 100 | 100 | 99 | 100 | 99 | 100 |
|  | 92 | 92 | 93 | 93 | 85 | 85 | 100 | 100 | 100 | 100 | 100 | 100 | 100 | 100 |
|  | 93 | 93 | 94 | 94 | 84 | 86 | 100 | 100 | 100 | 100 | 98 | 100 | 99 | 100 |
|  | 92 | 92 | 93 | 93 | 85 | 86 | 100 | 100 | 100 | 100 | 99 | 100 | 99 | 100 |
|  | 92 | 92 | 93 | 93 | 84 | 85 | 100 | 100 | 100 | 100 | 99 | 100 | 99 | 100 |
| RH | 81 | 81 | 93 | 93 | 89 | 88 | 100 | 100 | 100 | 100 | 100 | 99 | 100 | 99 |
|  | 78 | 78 | 93 | 93 | 92 | 92 | 100 | 100 | 100 | 100 | 100 | 100 | 100 | 100 |
|  | 83 | 83 | 93 | 93 | 89 | 90 | 100 | 100 | 100 | 100 | 99 | 100 | 99 | 100 |
|  | 67 | 67 | 93 | 93 | 92 | 93 | 100 | 100 | 100 | 100 | 99 | 100 | 99 | 100 |
|  | 76 | 76 | 93 | 93 | 92 | 92 | 100 | 100 | 100 | 100 | 100 | 100 | 100 | 100 |
|  | 58 | 58 | 92 | 92 | 92 | 92 | 100 | 100 | 100 | 100 | 100 | 100 | 100 | 100 |
|  | 73 | 73 | 87 | 92 | 93 | 94 | 100 | 100 | 95 | 100 | 99 | 100 | 97 | 100 |
|  | 77 | 77 | 91 | 91 | 91 | 92 | 100 | 100 | 100 | 100 | 99 | 100 | 99 | 100 |
|  | 61 | 61 | 91 | 91 | 94 | 94 | 100 | 100 | 100 | 100 | 100 | 100 | 100 | 100 |
|  | 63 | 63 | 92 | 92 | 93 | 93 | 100 | 100 | 100 | 100 | 100 | 100 | 100 | 100 |

DFI: Digital Facial Index; FH: forehead; E: eyes; M: mouth; L/R (subscript): left/right; JT: Johannes Taeger; SB: Stefanie Bischoff; KR: Kristen Rak; RH: Rudolf Hagen
